# P-175. Assessing the Dynamics of Fecal Colonization with Antibiotic-Resistant Bacteria in a Chilean Cohort: A Longitudinal Study

**DOI:** 10.1093/ofid/ofae631.380

**Published:** 2025-01-29

**Authors:** Araos Rafael, Alejandro Jara, Felipe Sanchez, Aiko Adell, Jorge Olivares, Catterina Ferreccio, Laura Huidobro, Macarena Garrido, Lina M Rivas, Anne S Peters, Jose M Munita, Erika M C D’Agata

**Affiliations:** Universidad del Desarrollo, Santiago, Region Metropolitana, Chile; Pontificia Universidad Católica de Chile, Santiago, Region Metropolitana, Chile; Pontificia Universidad Católica de Chile, Santiago, Region Metropolitana, Chile; Universidad Andrés Bello, Santiago, Region Metropolitana, Chile; Pontificia Universidad Católica de Valparaíso, Santiago, Region Metropolitana, Chile; Pontificia Universidad Católica de Chile, Santiago, Region Metropolitana, Chile; Universidad Católica del Maule, Santiago, Region Metropolitana, Chile; Pontificia Universidad Católica de Chile, Santiago, Region Metropolitana, Chile; Genomics & Resistant Microbes (GeRM), Instituto de Ciencias e Innovación en Medicina, Facultad de Medicina Clínica Alemana, Universidad del Desarrollo, Chile; Millennium Initiative for Collaborative Research on Bacterial Resistance (MICROB-R), Santiago, Region Metropolitana, Chile; Universidad del Desarrollo, Santiago, Region Metropolitana, Chile; Instituto de Ciencias e Innovación en Medicina, Santiago, Region Metropolitana, Chile; Brown University, Providence, Rhode Island

## Abstract

**Background:**

In low- and middle-income countries, the community burden of antibiotic-resistant Gram-negative bacteria (AR-GNB) colonization is high. Understanding factors influencing AR-GNB colonization is crucial for controlling antimicrobial resistance.

**Methods:**

We conducted a cohort study from December 2019 to November 2020 with community-dwelling adults in Molina, an agricultural town in Central Chile. We collected questionnaires and fecal samples every two weeks for six months to identify colonization by extended-spectrum cephalosporin-resistant Enterobacterales (ESCRE). ESCRE detection involved selective media screening, MALDI-TOF identification, and disk-diffusion antibiotic-susceptibility testing. We assessed the impact of epidemiological and clinical features and exposure to various water sources, foods, and animals on i) baseline ESCRE fecal colonization, ii) ESCRE acquisition during follow-up, and iii) ESCRE clearance. The study included 198 subjects, using multiple imputations for missing predictor values. We used two hidden Markov models for each dataset, with Model 1 assuming perfect sensitivity and specificity and Model 2 estimating sensitivity. A Bayesian approach with Markov chain Monte Carlo algorithms estimated odds ratios (OR) and credible intervals (CI).

**Results:**

The sample consisted of 198 residents with a mean age of 60.9 and a predominance of women (75%). Model 1 showed that treating drinking water before consumption reduced the risk of baseline ESCRE colonization (OR: 0.38; 95% CI: 0.02 – 0.89). No significant associations were found for ESCRE acquisition during follow-up. Males and individuals with comorbidities had a lower risk of clearing ESCRE (ORs: 0.58, 0.54; CIs: 0.24 – 0.97, 0.258 – 0.88, respectively). Model 2, with imperfect sensitivity, showed similar results.

**Conclusion:**

This study highlights the significance of environmental and individual health factors in ESCRE colonization. Interventions like treating drinking water could reduce baseline ESCRE colonization risk. The varied impacts of gender and comorbidities on ESCRE clearance provide insights for targeted AR-GNB management strategies. Further research is needed to understand these associations and develop effective community-level interventions against AMR.

**Disclosures:**

**All Authors**: No reported disclosures

